# Supplementary material for: The impact of subunit type, alternative splicing, and auxiliary proteins on AMPA receptor trafficking
Source: J Biol Chem. 2025 Apr 30;301(6):108569. doi: 10.1016/j.jbc.2025.108569 (PMC12152890; doi:10.1016/j.jbc.2025.108569)
Supplement: Supporting Information [file mmc1.docx]

**Supporting Information**


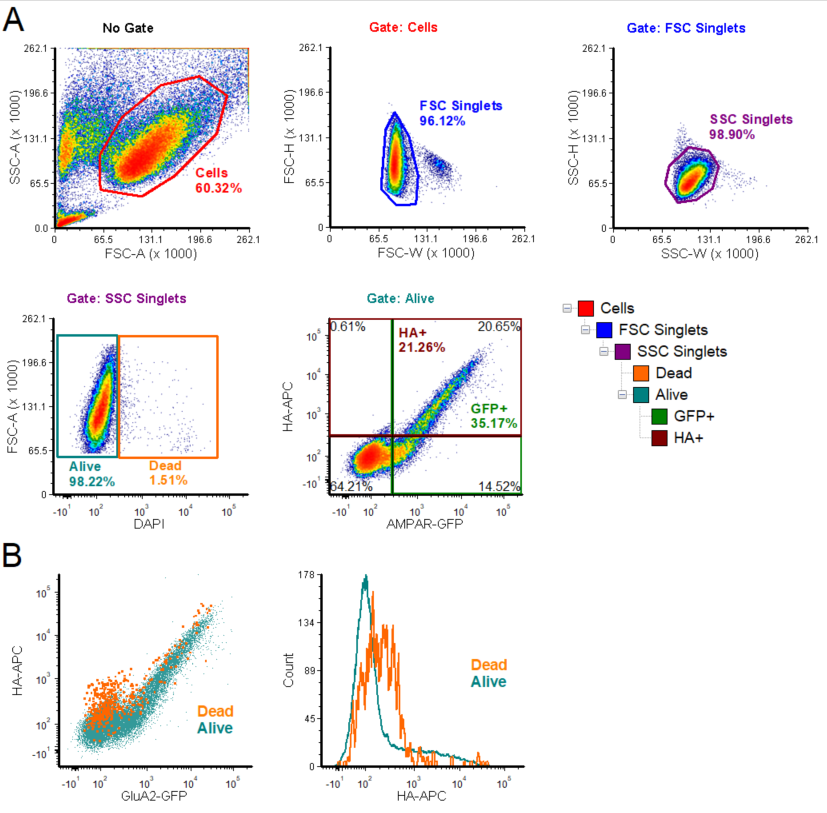


**Figure S1. Gating scheme with DAPI stain to remove false positive ‘surface’ signal. (A)** Gating scheme with forward and side scatter (FSC and SSC, respectively) gates as well as DAPI positive exclusion gate and quadrant plot. **(B)** Example GFP versus APC scatter (*left*) and APC histogram (*right*). DAPI positive cells (labelled Dead) are shown in orange and excluded cells in teal.


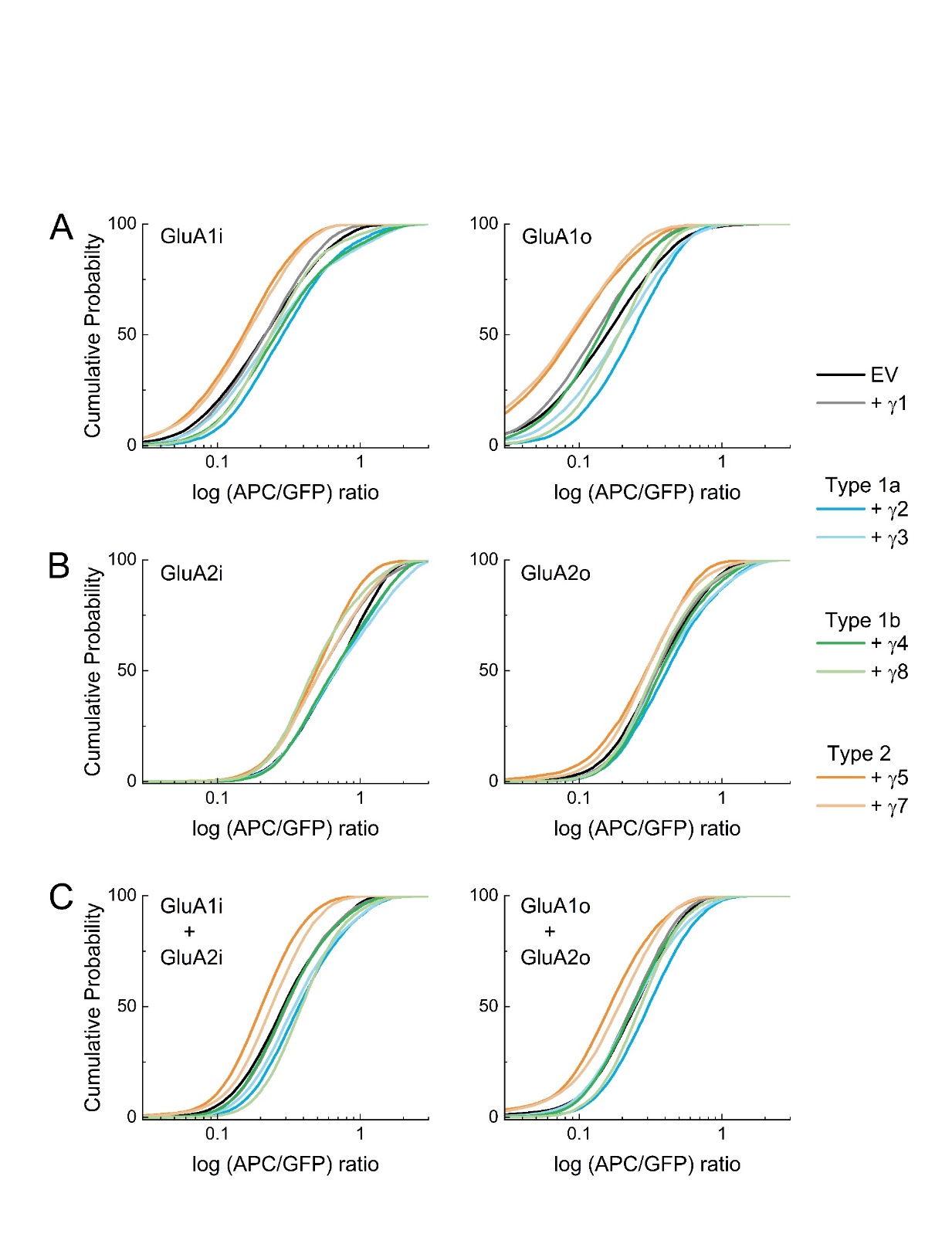


**Figure S2. Type 1 TARPs enhance while Type 2 TARPs impair surface trafficking of GluA1 and GluA2 AMPA receptors, both flip and flop. (A-C)** Single cell APC/GFP ratio cumulative probability plots for GluA1 **(A)**, GluA2 **(B)** and GluA1+GluA2 **(C)**, both flip (*left*) and flop (*right*) variants co-transfected with the indicated gamma subunits.


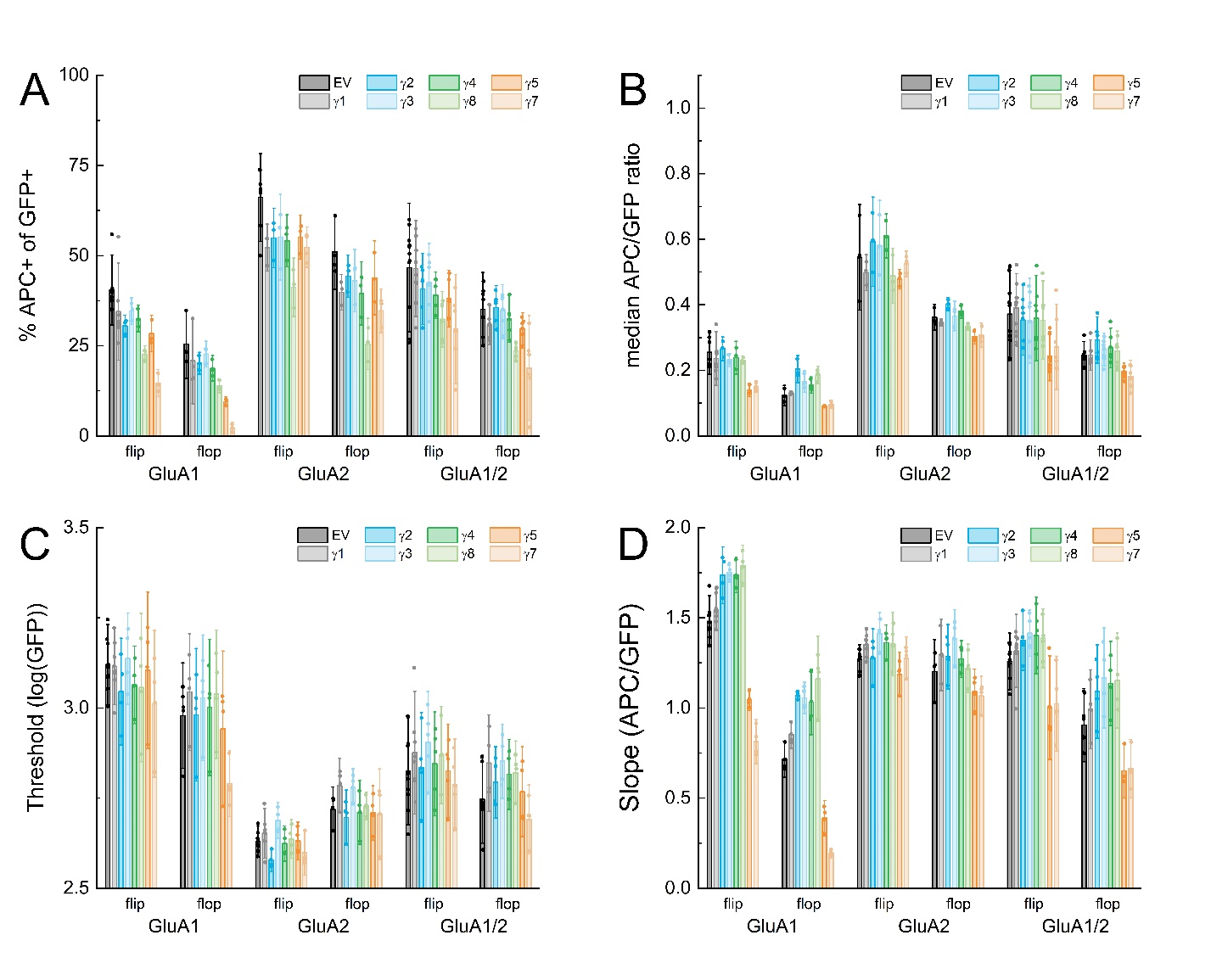


**Figure S3. Type I and 2 TARPs have opposing positive and negative effects on surface trafficking of GluA1 and GluA2 AMPA receptors, both flip and flop. (A, B)** Summary plots of percentage APC positive events of GFP positive events **(A)** and single-cell APC/GFP ratios **(B)**. Symbols show the median value from a single flow experiment, and column height and error bars show the mean and SD across flow experiments. **(C, D)** Summary of piecewise linear fit thresholds **(C)** and slopes **(D)** across flow experiments. Symbols indicate the fit value from individual flow experiments, while column heights and error bars represent the mean and SD across experiments.


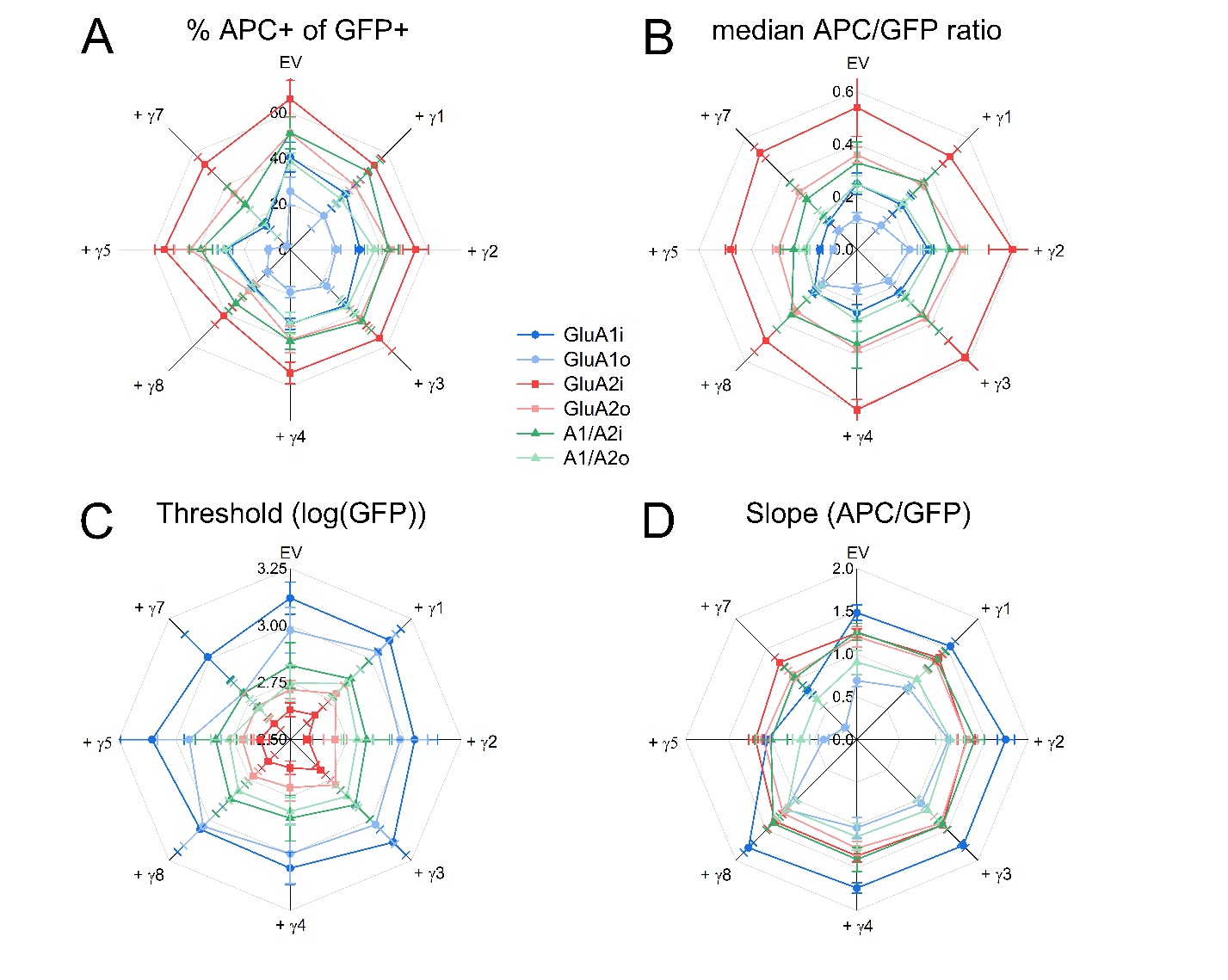


**Figure S4. Type I and 2 TARPs have opposing positive and negative effects on the surface trafficking of GluA1 and GluA2 AMPA receptors, both flip and flop. (A, B)** Radar plots of percentage APC positive events of GFP positive events **(A)** and median APC/GFP single cell ratios **(B)**. **(C, D)** Summary radar plots of thresholds **(C)** and slopes **(D)** across flow experiments. Symbols and error bars show the mean and SD, respectively, across flow experiments.


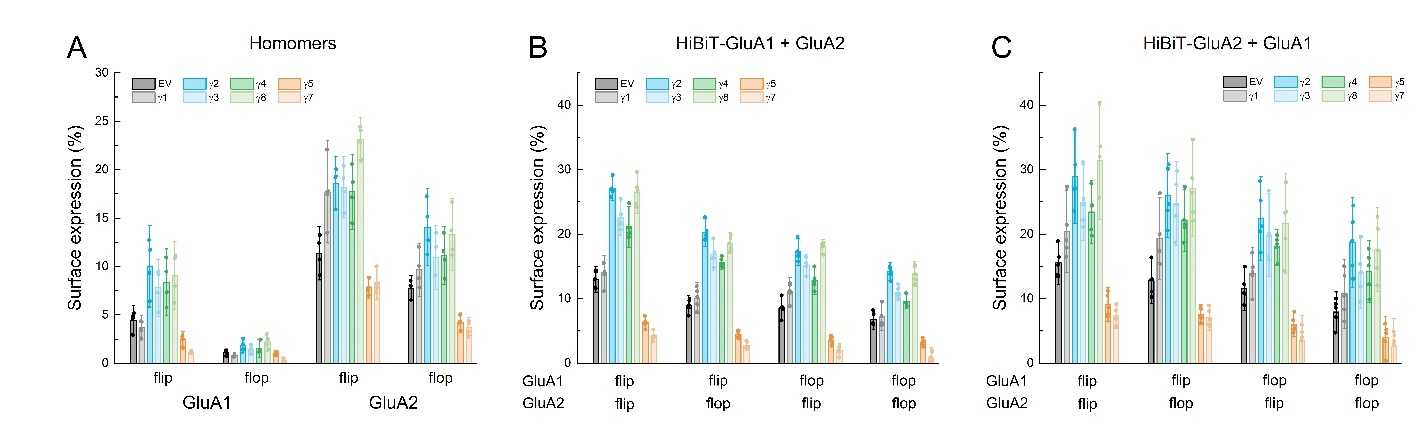


**Figure S5. TARPs impact both homomeric and heteromeric surface expression. (A)** Summary plot of AMPA receptor surface expression for GluA1 or GluA2, either flip or flop variant, when co-transfected with the individual TARP. **(B)** Summary plot of GluA1 surface expression when co-transfected with GluA2 and the indicated TARP. **(C)** Same as in **B** but for GluA2. Symbols show individual transfections and error bars are SD.


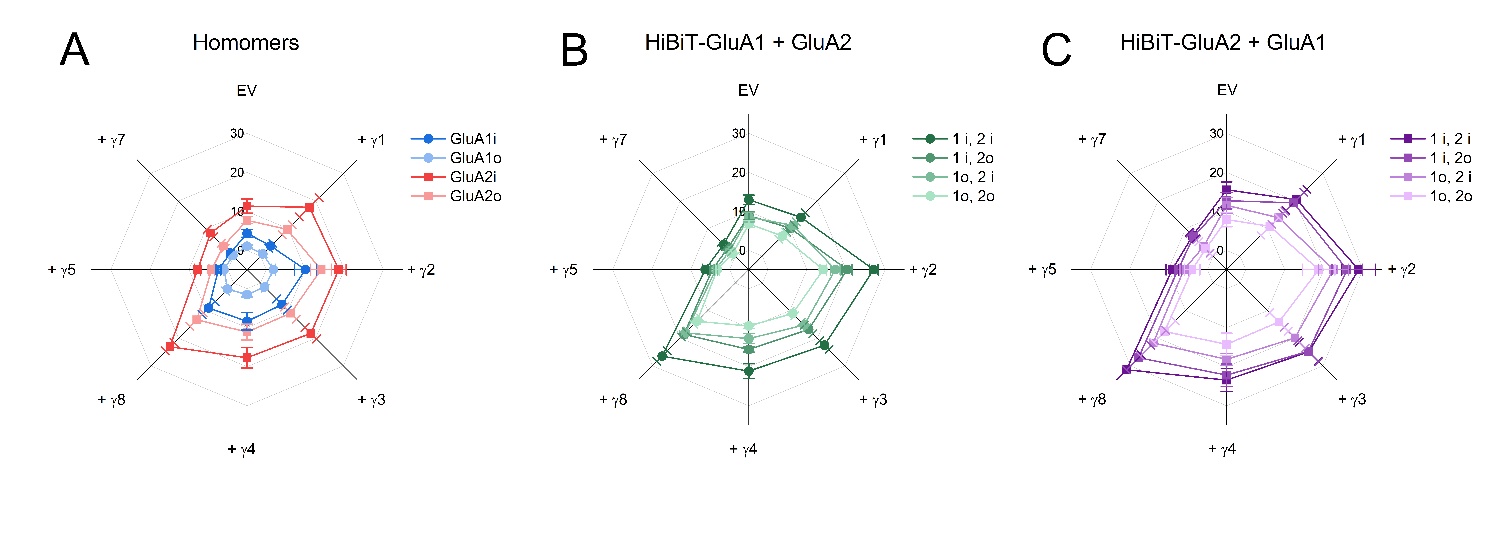


**Figure S6. Radar plots of TARP impact on homomeric and heteromeric surface expression. (A)** Radar plot of AMPA receptor surface expression for GluA1 or GluA2, either flip or flop variant, when co-transfected with the individual TARP. **(B)** Summary plot of GluA1 surface expression when co-transfected with GluA2 and the indicated TARP. **(C)** Same as in **B** but for GluA2. Symbols show mean of independent experiments and error bars are SD. Flip and flop are represented by *i* and *o*, respectively.


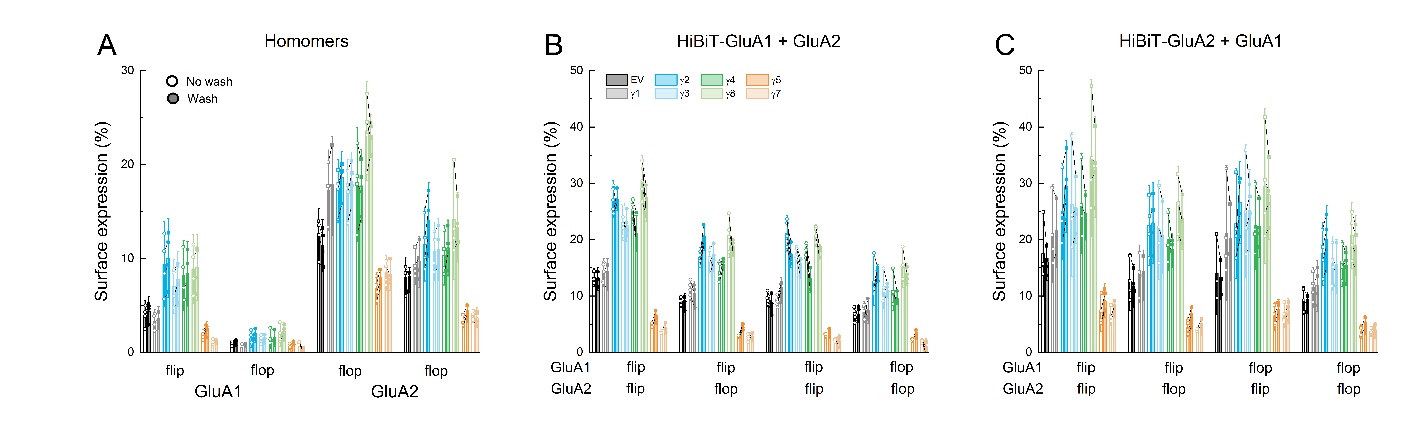


**Figure S7. Washing has negligible impact on surface expression results. (A-C)** Summary of surface expression for homomeric GluA1 or GluA2 **(A)**, or GluA1 **(B)** or GluA2 **(C)** when co-transfecting both subunits. Open symbols and bars are results from cells directly from growth media. Filled symbols are following wash steps. Symbols show individual experiments connected by lines and error bars are SD.
